# Evaluation of design, mechanical properties, and torque/force generation of heat-treated NiTi glide path instruments

**DOI:** 10.1186/s12903-022-02575-7

**Published:** 2022-11-24

**Authors:** Soram Oh, Ji-Yeon Seo, Ji-Eun Lee, Hyun-Jung Kim, Ji-Hyun Jang, Seok Woo Chang

**Affiliations:** 1 Department of Conservative Dentistry, Kyung Hee University Medical Center, 23 Kyungheedae-Ro, Dongdaemun-Gu, Seoul, 02447 Republic of Korea; 2 Department of Conservative Dentistry, Kyung Hee University College of Dentistry, 23 Kyungheedae-Ro, Dongdaemun-Gu, Seoul, 02447 Republic of Korea; 3 Private Dental Clinic, Seoul, Republic of Korea; 4 Seoul Strong Dental Clinic, 35 Bonghwasan-Ro, Jungnang-Gu, Seoul, 02017 Republic of Korea

**Keywords:** Heat treatment, Glide path, Mechanical test, NiTi instrument, Screw-in force, Torque

## Abstract

**Background:**

Recently, various kinds of heat-treated nickel-titanium (NiTi) glide path instruments have been manufactured. This study aimed to investigate design, phase transformation behavior, mechanical properties of TruNatomy Glider (#17/02), V Taper 2H (#14/03), and HyFlex EDM (#15/03) and compare torque/force generated during simulated glide path preparation with them.

**Methods:**

The designs and phase-transformation behaviors of the instruments were examined via scanning electron microscopy (*n* = 3) and differential scanning calorimetry (*n* = 2). Their bending (*n* = 15), torsional (*n* = 15), and cyclic fatigue resistances (*n* = 15) were tested. The ultimate strength and distortion angle were obtained from torsional resistance test. The number of cycles to failure (NCF) was calculated from cyclic fatigue resistance test. The preparation of the glide path was simulated using a double-curved artificial canal (*n* = 15), and the maximum torque and screw-in forces were measured. Data except NCF was compared between brands with one-way ANOVA with Tukey’s honestly significant difference test. NCF was analyzed via Kruskal–Wallis and Mann–Whitney U tests.

**Results:**

TruNatomy Glider had the greatest number of threads. TruNatomy Glider showed progressive taper, while V Taper 2H and HyFlex EDM had constant taper. The austenitic transformation-finish temperatures of all the instruments were above body temperature. V Taper 2H demonstrated significantly lower ultimate strength, higher distortion angle, and a higher number of cycles to failure compared with HyFlex EDM and TruNatomy Glider (*p* < 0.05). The maximum torque generated during preparing glide path was lowest for V Taper 2H, and the maximum screw-in force was lowest for HyFlex EDM (*p* < 0.05). TruNatomy Glider generated the highest torque and screw-in force during the apical preparation.

**Conclusions:**

V Taper 2H #14/03 showed superior cyclic fatigue resistance and lower ultimate strength. TruNatomy Glider generated greater clockwise torque and screw-in force during apical preparation. The mechanical properties, torque, and screw-force was affected by design of heat-treated glide path instruments. Cervical pre-flaring prior to glide path instrument is recommended.

## Background

The endodontic glide path is an open pathway from the coronal orifice to the apical limit of the root canal [1]. Glide path preparation employing nickel–titanium (NiTi) rotary instruments produces less transportation than hand instruments, reducing the stresses generated by subsequent shaping NiTi instruments [2, 3].

Despite increased efficiency and better centering ability of NiTi instruments, fracture during root canal shaping has been a primary concern to clinician [4]. The fractures of the NiTi instruments were caused by two mechanisms: the torsional and cyclic fatigue fractures [5]. Torsional fracture occurs when the tip of the instrument is tightly attached to the root canal while the shaft rotates continuously, while cyclic fatigue fracture originates from the repetitive stress due to the compression and tension in the curved root canal [6]. Heat-treated NiTi glide path instruments showed superior resistance to cyclic fatigue fracture compared to conventional NiTi instruments [7–9]. Inan et al. [10] reported higher ultimate strength and angular distortion from a heat-trated NiTi glide path instrument compared to those recorded from a conventional one. Nishijo et al. [11] reported higher ultimate strength for a heat-treated NiTi glide path instrument; however, the distortion angle of the heat-treated glide path instrument was lower than that of conventional one.

Mechanical properties, such as bending, torsional and cyclic fatigue resistances are affected by design of the instruments employed [12]. NiTi instruments with increased core mass and square cross-section, compared to those with triangular cross-section, had higher torsional resistance [10, 13]. NiTi glide path instruments with shorter pitch length showed increased ultimate strength and cyclic fatigue resistance [9, 14]. According to Kwak et al. NiTi glide path instruments with shorter pitch length showed smaller distortion angle [9], whereas Al Raeesi et al. reported no significant difference in distortion angle between two NiTi instrument groups with the same cross-sectional design and alloy but different pitch lengths [14]. Screw-in force refers to apically directed force generated when an instrument is screwed into a canal. NiTi glide path instrument with a greater taper generated higher maximum screw-in force [11]. The effect of the progressive taper of NiTi glide path instrument on the force/torque generation is still lacking.

A controlled memory (CM) wire, produced via heat treatment, comprises a certain amount of martensite at room temperature [15, 16]. NiTi instruments with CM wire present reduced superelasticity; therefore, they can retain modified form after external force was removed [5, 17]. V Taper 2H (SS White Dental, Lakewood, NJ, USA), which is manufactured from a CM wire, exhibits a regressive taper extending from the tip to shaft areas. Minimally invasive root canal shaping can be established by exploiting the regressive taper and slim wire in V Taper 2H [18]. TruNatomy (Dentsply Sirona, Ballaigues, Switzerland) has been introduced as a suitable instrument for conducting minimally invasive endodontic treatments [19]. Similar to V Taper 2H, the main features of TruNatomy include heat treatment, as well as the utilization of a slim wire and regressive taper of shaping instruments [20]. The TruNatomy Glider is used prior to the shaping instruments and exhibits a progressive taper and tip size of #17 [21]. Lastly, HyFlex EDM (Coltene/Whaledent AG, Altstätten, Switzerland) is produced via the electrical discharge machining of a CM wire [22]. Unlike the mechanical cutting process, electrical discharge machining is a shaping method that employs a spark that is generated between the workpiece and the tools [22]. HyFlex EDM exhibits superior resistance to cyclic fatigue, and its improved centering ability has also been reported [23, 24]. HyFlex EDM comprises two types of glide path instruments, namely #10/05 and #15/03, wherein the latter is typically used in severely curved canals [25].

Currently, no studies have investigated in detail the design, mechanical properties, and force/torque generation of the TruNatomy Glider #17/02, V Taper 2H #14/03, and HyFlex EDM #15/03 and compare with each other. Therefore, this study aimed to evaluate the designs of three types of NiTi glide path instruments, investigate their mechanical and metallurgical properties, and the torques and screw-in forces generated during their simulated glide path preparations. The null hypotheses tested in this study were (i) there are no differences in the tested mechanical and metallurgical properties among three brands of glide path NiTi instruments; and (ii) there are no differences in the torques and screw-in forces generated during simulated glide path preparation with three brands of glide path NiTi instruments.

## Methods

A total of 195 TruNatomy Glider (#17/02) (TRN), V Taper 2H #14/03 (V2H), and HyFlex EDM #15/03 (EDM) were tested (*n* = 65 each).

### Instrument design

The TRN, V2H, and EDM instruments were examined using scanning electron microscopy (SEM; S-4700, Hitachi, Tokyo, Japan) at × 40 magnifications (*n* = 3 each). The number of threads, the helix angles, and the surface characteristics of the instruments were investigated using the SEM images. For the measurement of the helix angle, the angles between the longitudinal axis of the instrument and cutting edges located at distances of 3, 6, and 9 mm from the tip of the instrument were measured using ImageJ software (NIH, Bethesda, MD, USA).

### Differential scanning calorimetry (DSC)

DSC was performed by DSC250 (TA Instruments, New Castle, DE, USA) on tip 3 mm of unused TRN, V2H, and EDM (two samples per brand). The samples were first heated from 25 to 90 °C, then cooled to − 90 °C, and finally heated again to 90 °C at a heating rate of 10 °C/min. DSC was used to determine the associated energy changes and the martensitic, austenitic, and R-phase transformation-start and finish temperatures, given by M_s_, M_f_, A_s_, A_f,_ R_s,_ and R_f_, respectively.

### Mechanical tests

Sample size calculation was performed using a *priori* ANOVA (fixed effects, omnibus, one-way) with G*Power for Windows v3.1 (Heinrich Heine, Universität Düsseldorf, Düsseldorf, Germany), using an alpha type error of 0.05 and a power beta of 0.85. The effect size was calculated (2.04) from the results of a previous study with similar methodology [10]. A minimum of 9 samples were required per group to observe the same effect. Each of the 15 instruments from TRN, V2H, and EDM were subjected to three mechanical tests: bending, torsional and cyclic fatigue resistance. All NiTi instruments were examined with a dental operating microscope (G3; Global, St. Louis, MO, USA) at a 12.8 magnification to detect any deformity, and none of the samples were discarded.

Bending, torsional, and cyclic fatigue resistance tests were conducted at 22℃ to evaluate the mechanical properties of the TRN, V2H, and EDM. The bending and torsional fracture resistances of the instruments were tested according to ISO 3630–1. A universal testing machine (UTM; Universal Mechanics Analyzer, IB Systems, Seoul, Korea) connected to a notebook was used for the tests. The UTM comprised of an upper part, consisting of stainless-steel jaws operated by a reversible geared motor, and a lower part connected to a load cell and torque sensor. To test the bending resistance, a NiTi instrument with a 3 mm tip was tightly clamped onto a chuck that was connected to the lower part of the UTM. The NiTi instrument was rotated in the clockwise direction at 2 rpm using a metal rod connected to the upper part of the UTM. The bending resistance (Ncm) was recorded as the torque required to displace the instrument at 45°. To test the torsional resistance, the tip of the NiTi instrument (3 mm) was clamped onto a lower chuck with brass jaws, then the instrument's shaft was fixed to the upper chuck and rotated continuously in the clockwise direction at 2 rpm until fracture. The ultimate strength (Ncm) and distortion angle (°) at fracture were recorded for statistical analysis.

To test the cyclic fatigue resistance, static test was conducted using an artificial root canal produced from stainless steel. The artificial canal had an inner diameter of 1 mm, and the curvature exhibited a 1.5-mm radius and 60° angle, as determined by Pruett’s method [26]. The instruments were rotated continuously at the manufacturers’ recommended speeds of 500, 300, and 300 rpm for TRN, V2H, and EDM, respectively. Lubricants were applied during the tests to prevent excessive heating. The mean time to failure was obtained and multiplied by rotational speed to calculate the number of cycles to failure (NCF). The fracture length of the instruments were measured.

The fractured instruments from the torsional and cyclic fatigue resistance tests were examined using SEM (S-4700 and Apreo S; Thermo Scientific, Waltham, MA, USA).

### Glide path preparation

The glide path was prepared on an artificial resin canal to ensure consistent anatomy. Forty-five double-curved resin blocks (Endo Training Blocks, Dentsply Sirona), with 0.1 mm apical diameter, 2% taper, and a working length of 16.5 mm were utilized. One instrument was used for every single canal (*n* = 15). Instrumentation was performed using an X Smart Plus (Dentsply Sirona), and the handpiece was firmly clamped to the UTM. The automatic vertical movement of the instruments was controlled using the UTM. The resin block was fixed to the lower part of the UTM to measure the torque and force during preparation of glide path in real-time. The simulated glide path preparation was performed in two parts:12.5 to 14.5 mm, i.e., from 4 to 2 mm short of the working length, and 14.5 to 16.5 mm, i.e., from 2 to 0 mm short of the working length. Each cycle consisted of three pecking motions, i.e., three repetitions of 2 mm-downward followed by 2 mm-upward movement. For shaping the first part, the NiTi instrument was inserted to up to 12.5 mm of its length. Three continuous dynamic pecking motions were performed up to 14.5 mm-depth at 1.17 mm/s. The sum of the vertical moving distances was 12 mm, therefore; therefore, it took approximately 10 s to complete one cycle. During the vertical movement, the instrument was rotated according to the recommended rpm: 500, 300, and 300 for TRN, V2H, and EDM, respectively. Afterward, the NiTi instrument was removed from the canal, the debris was wiped off, and the canal was irrigated with 1 ml of distilled water. Thereafter, the instrument was inserted at a length of 14.5 mm to shape the second part. Three continuous dynamic pecking motions were performed up to 16.5 mm-depth. For the three brands of instrument, the maximum clockwise torques (Ncm) generated when shaping ‘apical 2–4 mm’ region (Torque1) and ‘apical 0–2 mm’ region (Torque2) were obtained. Forces in upward direction was defined as the screw-in force, of which the peak values generated during shaping ‘apical 2–4 mm’ region (Force1) and ‘apical 0–2 mm’ region (Force2) were measured.

### Statistical analysis

The normal distribution of the mechanical test data was verified using the Shapiro–Wilk test, and the homogeneity of the variances (except that of NCF) was verified by Levene’s test. The bending resistance (Ncm), ultimate strength (Ncm), distortion angle (°), and fractured length (mm) were analyzed via one-way ANOVA with Tukey’s honestly significant difference (HSD) test to compare the three brands. NCFs were analyzed using Kruskal–Wallis and Mann–Whitney U tests.

The data of Torque1 and Torque2 (Ncm), as well as Force1 and Force2 (gf), satisfied the normal distribution and homogeneity of variances; their values among the three brands of instrument were compared using one-way ANOVA and Tukey’s HSD test. Torque1 and Torque2 were compared using the paired t-test for each brand of NiTi instrument; Force1 and Force2 were also compared.

## Results

### Instrument design

**Fig. 1:**
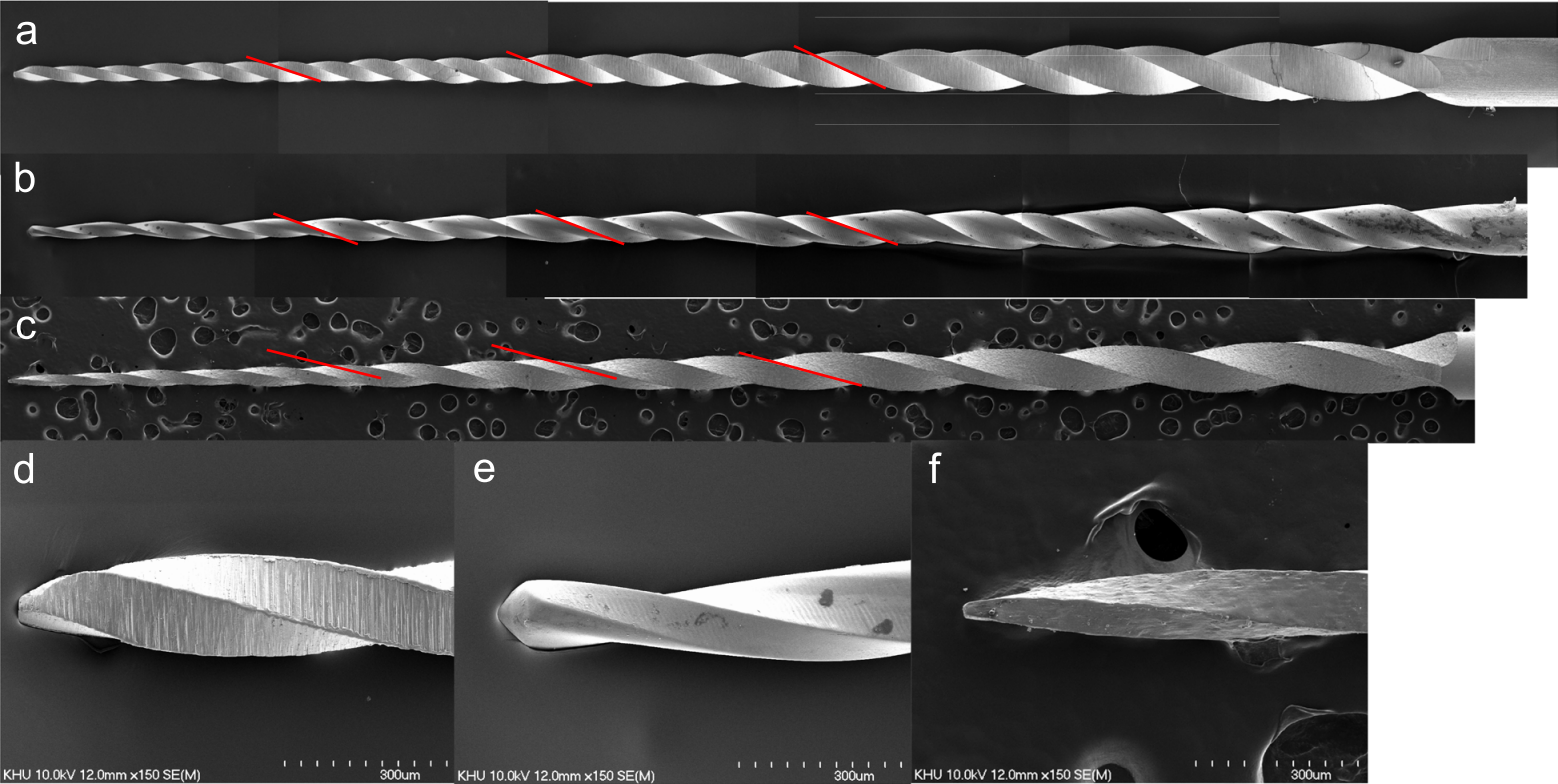
SEM images of TruNatomy Glider, V Taper 2H, and HyFlex EDM. Lateral surfaces of the TruNatomy Glider (**a**), V Taper 2H (**b**), and HyFlex EDM (**c**). Their helix angles were measured at cutting edges around 3, 6, and 9 mm from the tip (red lines); The tip areas of TruNatomy Glider (**d**), V Taper 2H #14/03 (e), and HyFlex EDM #15/03 (f)

The lateral images of the new instruments revealed that the tapers of TRN increased from the apical region to the coronal region. In contrast, V2H and EDM exhibited a constant taper (Fig. 1). TRN exhibited the highest number of threads (24), followed by V2H (20) and EDM (15). The helix angles for TRN were 19.2°, 20.7°, and 24.1°, for V2H were 19.7°, 20.4°, and 22.6°; and for EDM were 11.9°, 13.4°, and 19.0° in the 3-, 6-, and 9-mm regions, respectively. TRN and EDM exhibited variable pitches, which increased gradually towards the shaft (Figs. 1a and c). The longitudinal design of V2H comprised repetitions with two wide flutes and one narrow one; a unit of the repeating three flutes widened towards the shaft (Fig. 1b). Machining grooves were detected on the lateral surfaces of TRN and V2H (Fig. 1d and e). EDM exhibited a crater-like surface without a machining groove (Fig. 1f).

### Differential scanning calorimetry

**Fig. 2:**
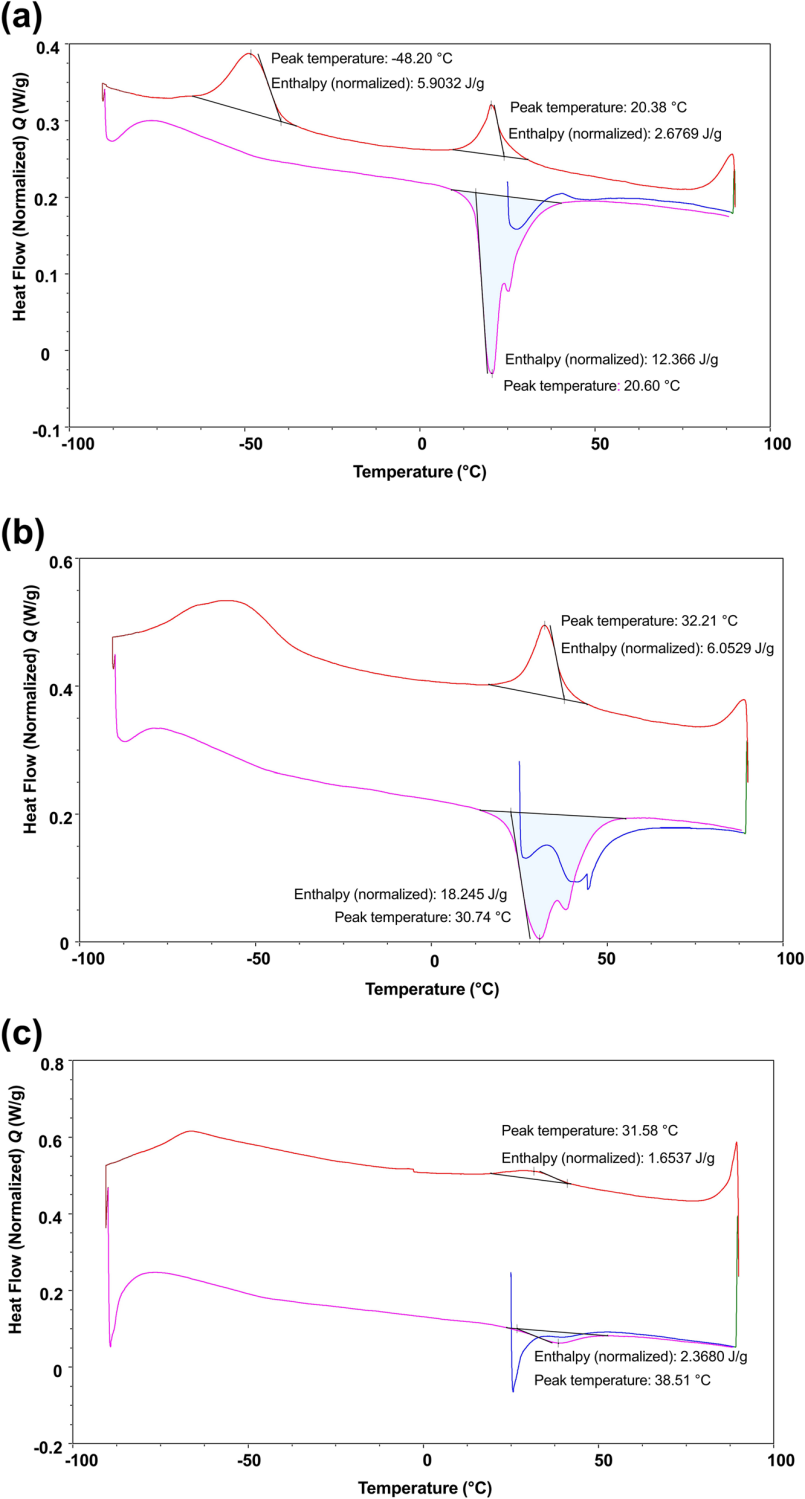
DSC curves of the unused TruNatomy Glider (**a**), V Taper 2H (**b**), and HyFlex EDM (**c**)

The heating curves of the tested instruments exhibited a single curve, indicating austenitic transformation (Fig. 2). The cooling curves of V2H and EDM exhibited single curves corresponding to martensitic transformations (Figs. 2b and c). The cooling curve of TRN exhibited two peaks, indicating the occurrence of an R-phase transformation, followed by a martensitic transformation (Fig. 2a). The transformation temperatures and enthalpy changes were determined from DSC curves (Table 1). The A_f_ values of TRN, V2H, and EDM were 40.60 °C, 55.0 °C, and 52.54 °C, respectively.

**Table 1:** Transformation temperatures and associated energies obtained from the DSC plots

	**Cooling**						**Heating**		
	R_s_ (°C)	R_f_ (°C)	ΔH (J/g)	M_s_ (°C)	M_f_ (°C)	ΔH (J/g)	A_s_ (°C)	A_f_ (°C)	ΔH (J/g)
**TruNatomy Glider**	31.68	8.92	2.68	− 35.43	− 63.63	5.90	8.93	40.60	12.37
**V Taper 2H**				44.23	15.38	6.05	13.46	55.0	18.25
**HyFlex EDM**				41.54	18.80	1.65	25.85	52.54	2.37

### Mechanical tests

**Fig. 3:**
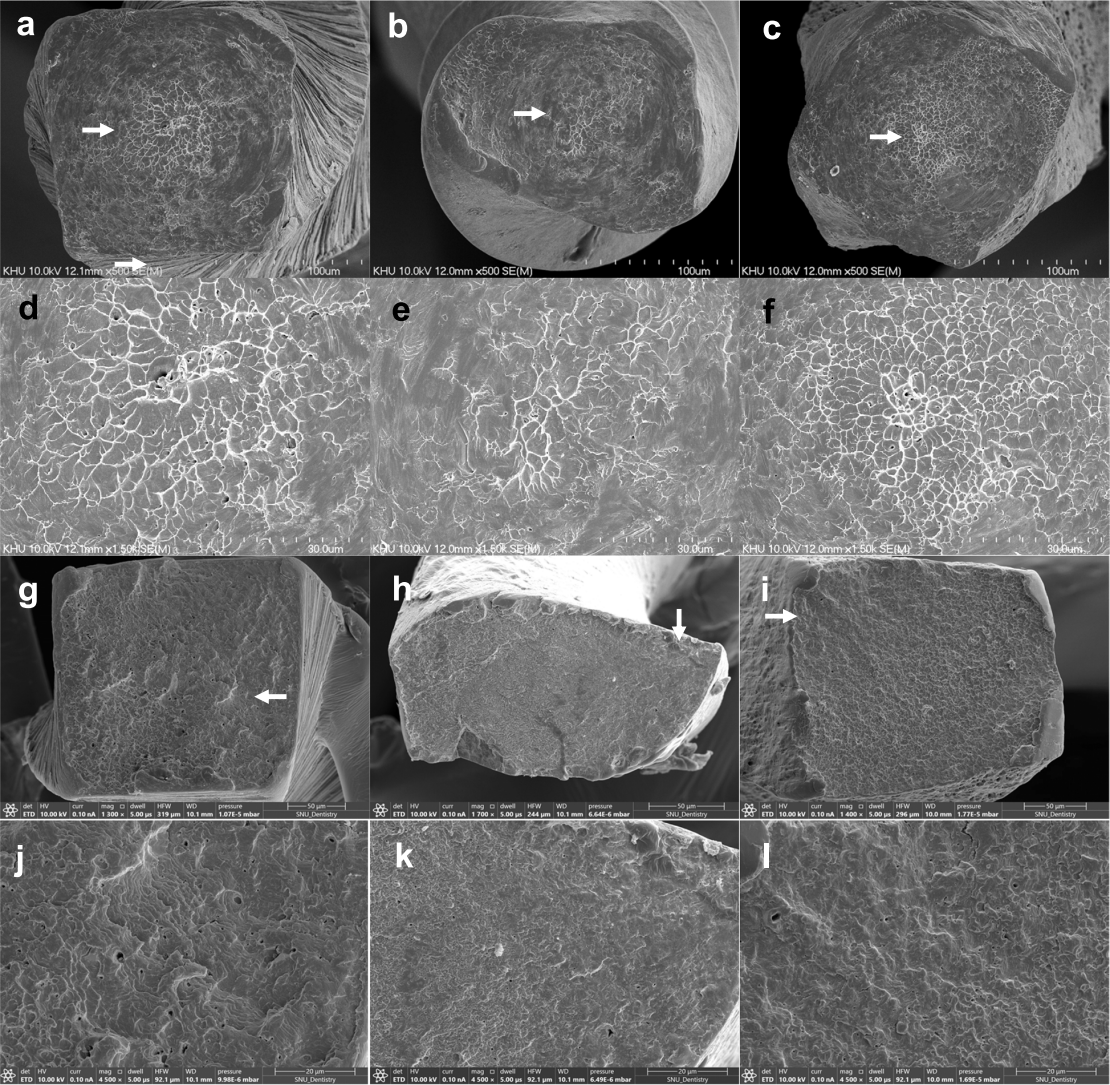
SEM images of the fractured instruments. Fractured surfaces of TruNatomy Glider (**a**), V Taper 2H (**b**), and HyFlex EDM (**c**) from torsional resistance test. (**d**–**f**) Magnified images of white arrows in (**a**–**c**). Fractured surfaces of TruNatomy Glider (**g**), V Taper 2H (**h**), and HyFlex EDM (**i**) from the cyclic fatigue resistance test. (**j**–**l**) Magnified images of white arrows in (**g**–**i**)

The bending resistance of the three instrument groups was not significantly different (*p* > 0.05) (Table 2). V2H demonstrated significantly lower ultimate strength, a higher distortion angle, and higher NCF than TRN and EDM (*p* < 0.05), and those of the two instruments (TRN and EDM) were not significantly different (*p* > 0.05) (Table 2). No significant differences were observed between the fracture lengths of the NiTi instruments (*p* > 0.05). The SEM images of the fractured instruments from the torsional and cyclic fatigue resistance tests showed concentric abrasion marks around the center of rotation (Figs. 3a–f) and multiple fatigue striations, respectively (Figs. 3g–l).

**Table 2:** Results of the mechanical tests of the NiTi glide path instruments

	**Bending resistance (Ncm)**	**Torsional resistance**		**Cyclic fatigue resistance**	
NiTi files		**Ultimate strength (Ncm)**	**distortion angle (°)**	**NCF**	**Fractured length (mm)**
TruNatomy Glider	0.0913 (0.0213)	0.2674 (0.0729)^a^	592.168 (56.601)^a^	655.0 (77.8)^a^	3.87 (0.51)
V Taper 2H	0.0855 (0.0326)	0.1944 (0.08)^b^	832.664 (92.919)^b^	2220.67 (435.49)^b^	3.81 (0.81)
HyFlex EDM	0.0769 (0.0332)	0.2737 (0.0779)^a^	543.127 (75.853)^a^	648.67 (106.54)^a^	3.61 (0.75)

The different superscript letters indicate the significant differences among the brands of NiTi instrument (*p* < 0.05)

### Glide path preparation

**Fig. 4:**
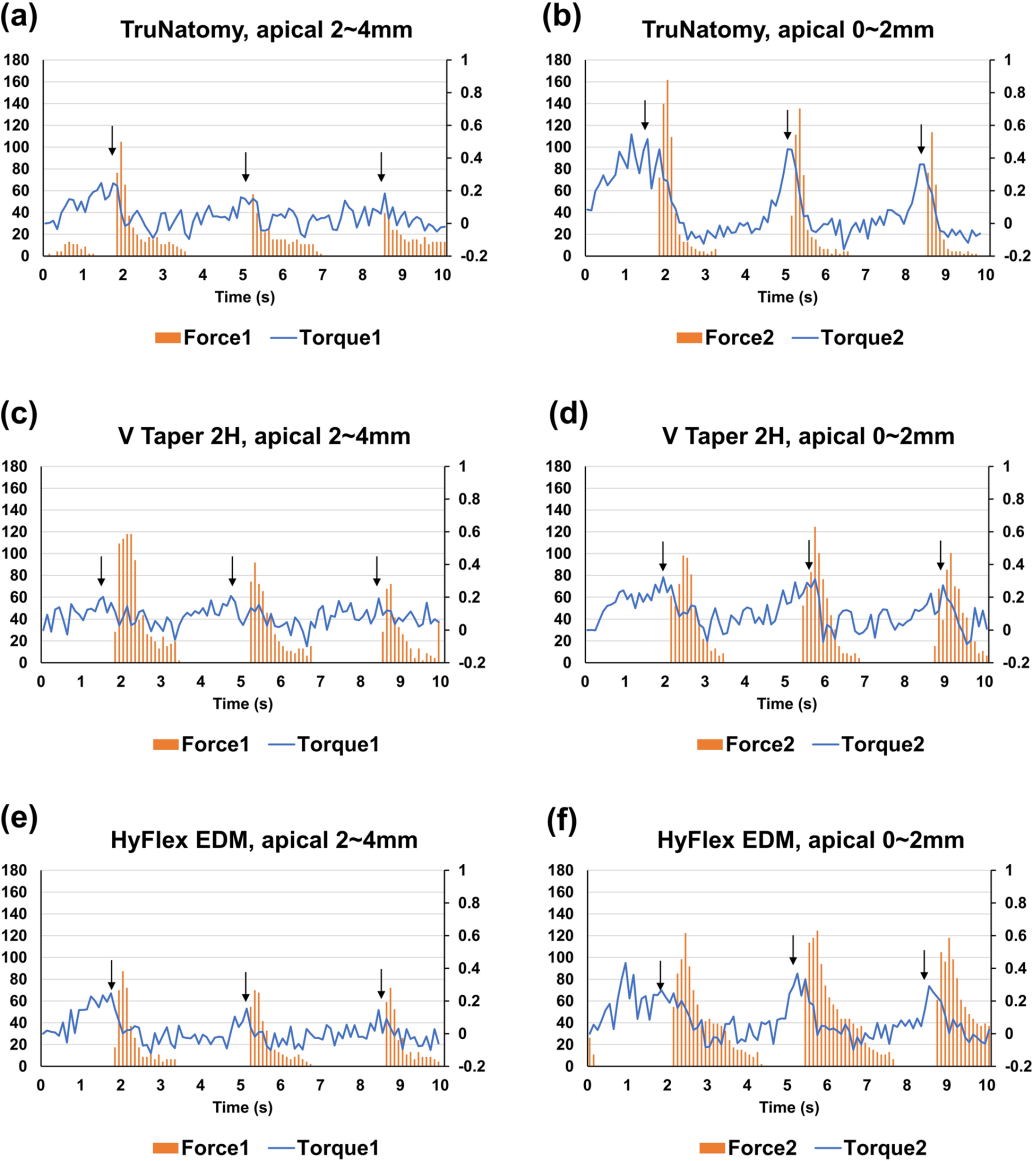
Representative graphs of the clockwise torque and screw-in forces that were generated during the simulated preparation of the glide path. Real-time clockwise torque and screw-in force generated by TruNatomy Glider during preparation of apical 2–4 mm and apical 0–2 mm, respectively (**a**, **b**); V Taper 2H during preparation of apical 2–4 mm and apical 0–2 mm, respectively (**c**, **d**); HyFlex EDM during preparation of apical 2–4 mm and apical 0–2 mm, respectively (**e**, **f**)

No instrument was fractured during the simulated preparation of glide path. The clockwise torque increased as the instrument moved downward (toward the apex) during the dynamic movement; the screw-in force showed a peak value immediately after the direction of the movement was switched from downward to upward (Figs. 4a–f). EDM exhibited a higher Torque1 than V2H (*p* = 0.006) (Table 3). TRN and EDM exhibited a higher Torque2 than V2H (*p* < 0.001) (Table 3). TRN and V2H exhibited higher Force1 than EDM (*p* = 0.002 and 0.018, respectively). TRN generated a higher Force2 than EDM and V2H (*p* < 0.001 and *p* = 0.002, respectively). For each brand of instrument, Torque2 and Force2 were higher than Torque1 and Force1, respectively (*p* < 0.05).

**Table 3:** Maximum clockwise torques, and maximum screw-in forces generated during glide path preparation. Mean (standard deviation)

	**Maximum clockwise torque (Ncm)**		**Maximum screw-in force (gf)**	
Shaping region	**Apical 2**–**4 mm**	**Apical 0**–**2 mm**	**Apical 2**–**4 mm**	**Apical 0**–**2 mm**
TruNatomy Glider	0.209 (0.055)^a,b^	0.549 (0.115)^a^*	108.17 (21.249)^a^	154.21 (18.371)^a^†
V Taper 2H	0.181 (0.072)^b^	0.284 (0.093)^b^*	102.68 (20.153)^a^	128.14 (20.965)^b^†
HyFlex EDM	0.254 (0.057)^a^	0.465 (0.078)^a^*	81.765 (18.762)^b^	121.5 (17.959)^b^†

The different superscript letters within each column indicate significant differences among the brands of NiTi instrument (*p* < 0.05)

^*^ The maximum clockwise torque generated during apical 2 mm was higher than that during apical 2–4 mm (*p* < 0.05)

^†^ The maximum screw-in force generated during apical 2 mm was higher than that during apical 2–4 mm (*p* < 0.05)

## Discussion

The TRN brand had the greatest number of threads and an increased taper while EDM exhibited the least number of threads. The V2H consisted of repetitions with two wide flutes and one narrow flute. Although TRN, EDM and V2H demonstrated austenitic transformation-finish temperature above 37℃, phase transformation behaviors were different among three brands of NiTi glide path instruments. The bending resistances of the three brands were not significantly different. V2H exhibited a significantly lower ultimate strength, higher distortion angle, and higher NCF than those of TRN and EDM, and those of the two instruments (TRN and EDM) were not significantly different. All three NiTi instrument brands were used safely in glide path preparation of curved resin canals without fracture. TRN and EDM exhibited higher maximum clockwise torque compared to V2H, and TRN generated a higher maximum screw-in force compared to EDM and V2H during the shaping of apical 2 mm region. Therefore, the null hypotheses were rejected.

The questions raised for this study were whether mechanical and metallurgical properties of the three brands of glide path NiTi instruments were different, and whether the torques and screw-in forces generated during simulated glide path preparation with the instruments were different. Moreover, this study aimed to investigate whether the design and metallurgical properties influence on the mechanical properties and the torque/force generation during simulated glide path preparation.

The pitch length was the shortest for TRN, followed by V2H and EDM. Although TRN and EDM had the shortest and longest pitch length, respectively, there was no significant difference in their mechanical properties. Our results disagree with those of previous studies that reported that instruments with shorter pitch lengths showed higher ultimate strength and superior cyclic fatigue resistance [9, 14]. All three instruments in this study have different designs, tip sizes and manufacturing methods, therefore, the effect of pitch length on torsional, and cyclic fatigue fracture resistances cannot be strictly determined. Nevertheless, EDM was not inferior to TRN in mechanical properties despite longer pitch length of EDM, because of the non-cutting method of manufacturing process.

The bending resistance of a NiTi instrument was related to heat treatment, which determined its phase composition and flexibility [27, 28]. EDM and V2H, known as CM-wire NiTi instruments, were mainly present in a martensitic phase. Whereas TRN consisted of a mixture of austenite, martensite, and R-phase at 22℃ (Fig. 2). The three tested glide path instruments had comparable bending resistances owing to the presence of martensite and/or the R-phase. The mean bending resistances recorded in this study ranged from 0.0769 to 0.0913 Ncm, which is similar to the result of heat-treated glide path instruments in a previous study [27].

V2H demonstrated the highest distortion angle and NCF while exhibiting the lowest ultimate strength. TRN, V2H, and EDM exhibited D3 values of 0.23, 0.23, and 0.24 mm, respectively. TRN and EDM had quadratic cross-sections (Figs. 3a, c, g, i), whereas V2H showed a trapezoidal or parabolic cross-sectional shape (Figs. 3b and h). The quadratic or rectangular cross-section withstood a higher maximum torque during the torsional resistance test, as observed in previous studies [10, 13]. Conversely, V2H rotated at a larger angle until torsional fracture occurred due to its reduced metal mass with a parabolic cross-section. Moreover, the longitudinal design of the V2H exhibited a characteristic alternating pattern of repetition with two wide flutes and a narrow one (Fig. 1b). This unique design might have contributed to relieving the stress during the torsional resistance test and allowing more deformation before fracture.

There is no international standard regarding the experimental setup for the cyclic fatigue resistance test of the NiTi instrument. In this study, the cyclic fatigue resistance was tested in a stainless-steel canal with an inner diameter of 1 mm. The instrument was allowed to rotate freely inside the canal. A 3.5 ~ 4 mm region from the tip of the instrument was placed in the curved part of the canal and exposed to tensile and compressive forces. In previous studies, more flexible NiTi instruments demonstrated superior resistance to cyclic fatigue [5, 29]. Because no significant differences existed among the bending resistances of the three instruments (Table 1), indicating flexibility, it was reasonable to conclude that the cross-sectional shape and longitudinal design contributed to cyclic fatigue resistance [30, 31]. The fractured cross-sectional area of V2H was smaller than those of TRN and EDM (Fig. 3g, h, i). V2H exhibited an alternating pitch length, and the contact area of the instrument was minimized during the cyclic fatigue resistance test. In this study, the cyclic fatigue resistance tests were performed at 22 ℃. Previous studies reported that cyclic fatigue resistances of conventional and the heat-treated NiTi instruments were reduced by testing at 37 ℃ compared to 20–22 ℃ [32–35]. Although all the three NiTi instruments tested in this study exhibited A_f_ values of ≥ 40 °C, phase transformation temperatures and enthalpy changes were different among three brands. Further study is needed to compare cyclic fatigue resistances at 37℃.

In the present study, glide path preparation was simulated using an S-shaped resin canal consisting of double curvatures; the first was at apical 3.5–5 mm, and the second was at apical 0–1 mm. V2H exhibited the lowest clockwise torque throughout the simulation (Table 3). It is speculated that the parabolic cross-sectional shape and reduced cross-sectional area accounted for the reduced torque. TRN exhibited an increased taper toward the shaft. The maximum diameter of the cutting edges of the TRN was greater than those of the V2H and EDM (Fig. 1). TRN generated the highest clockwise torque and screw-in force during apical preparation (Table 3), which correlates with the findings of a previous study that reported that instruments with large tapers generated higher torques and screw-in forces than those with narrow tapers [36]. All three brands of instrument, TruNatomy, V Taper 2H, and HyFlex EDM, consist of an orifice pre-flaring NiTi instrument, glide path preparation instrument, and shaping instruments, which are instructed to be used sequentially. In the present study, orifice pre-flaring NiTi instruments were not used, since they have different tip sizes and tapers. If cervical pre-flaring was performed prior to simulated glide path preparation, the torque and screw-in force would have decreased [37]. In addition, it is necessary to investigate the effects of various glide path instruments on the torque/force generated when shaping NiTi instruments are followed.

EDM resulted in the lowest screw-in force during the simulated preparation of the glide path. Among the three NiTi instruments, the EDM exhibited the highest pitch length and variable pitch. Conversely, a previous finite element analysis reported that instruments with smaller pitches generated less screw-in force than those with higher pitches [38]. The constant taper of EDM probably contributed more to the reduction of the screw-in force compared with the progressively increasing tapers of TRN. Irrespective of the instrument brand, the torques and screw-in forces generated during the shaping of the ‘apical 0–2 mm’ region (Torque2, Force2) were higher than those of the ‘apical 2–4 mm’ region (Torque1, Force1) (Table 3). Because the instrument was inserted closer to the apex, the contact area between the instrument and the canal wall was widened, increasing the torque and screw-in forces [36].

This study investigated characteristics of TRN, V2H, and EDM using a multi-method approach. Numerous studies have compared the mechanical properties of NiTi instruments without inspection of design or phase transformation behavior. Conversely, this study provides comprehensive information to understand the three brands of NiTi glide path instruments. All three brands of NiTi instruments generated higher clockwise torque and screw-in force during apical 2 mm preparation than during apical 2-4 mm preparation. Cervical pre-flaring prior to the use of glide path instrument is recommended. The limitation of this study is that the mechanical tests were performed at 22℃. Although a previous study reported that torsional resistance of four brands of NiTi files did not differ according to the test temperature [39], the cyclic fatigue and bending resistances were affected by temperature [28, 32, 33]. Further studies are needed for conduct of bending, torsional and cyclic fatigue resistance tests at body temperature. Another limitation of this study was the use of an artificial resin block, instead a human tooth. Because the physical properties of resin blocks and human teeth, such as strength and hardness, differ, the results of this study cannot be extrapolated directly to clinical practice. Additional studies are required on the generation of torque and force during the preparation of root canals of human teeth by glide path preparation employing different NiTi glide path instruments.

## Conclusions

The design of heat-treated NiTi glide path instruments influenced the clockwise torque and screw-in force generated during glide path preparation, as well as mechanical properties. V Taper 2H #14/03 presented the highest distortion angle and cyclic fatigue resistance, while its ultimate strength was the lowest. The NiTi glide path instrument with a progressive taper generated greater clockwise torque and screw-in force during apical preparation. Pre-flaring of root canal orifice prior to use of the glide path instrument is recommended.

## Data Availability

The datasets used and/or analysed during the current study are available from the corresponding author on reasonable request.

## Abbreviations

NiTi: Nickel–titanium
CM: Controlled memory
TRN: TruNatomy Glider
V2H: V Taper 2H #14/03
EDM: HyFlex EDM #15/03
SEM: Scanning electron microscopy
DSC: Differential scanning calorimetry
M_s_: Martensitic transformation-start temperature
M_f_: Martensitic transformation-finish temperature
A_s_: Austenitic transformation-start temperature
A_f_: Austenitic transformation-finish temperature
R_s_: R-phase transformation-start temperature
R_f_: R-phase transformation-finish temperature
UTM: Universal testing machine
NCF: Number of cycles to failure
Torque1: The maximum clockwise torque generated when shaping ‘apical 2–4 mm’ region
Torque2: The maximum clockwise torque generated when shaping ‘apical 0–2 mm’ region
Force1: Peak value of the screw-in force generated during shaping ‘apical 2–4 mm’ region
Force2: Peak value of the screw-in force generated during shaping ‘apical 0–2 mm’ region
